# The Effect of Five Different Wetting Treatments on the Nutrient Content and Microbial Concentration in Hay for Horses

**DOI:** 10.1371/journal.pone.0114079

**Published:** 2014-11-26

**Authors:** Meriel Jean Scott Moore-Colyer, Kimberly Lumbis, Annette Longland, Patricia Harris

**Affiliations:** 1 Royal Agricultural University, Cirencester, Gloucestershire, United Kingdom; 2 Equine and Livestock Nutrition Services, Pantafallen Fach, Tregaron, Ceredigion, Wales; 3 Mars Horsecare UK LTD; Equine Studies Group, Waltham Centre for Pet Nutrition, Freeby Lane, Waltham-on-the Wolds, Leicestershire, United Kingdom; Graz University of Technology (TU Graz), Austria

## Abstract

Five different hays were used to determine the effect of 5 different soaking and steaming treatments on the water soluble carbohydrate and microbial (bacteria and mould) contents of UK hay. Hays were subjected to the following 5 treatments: 1. Dry; 2. Steamed for 50 minutes in the Haygain- 600 steamer; 3. Soaked in water at 16°C for 9 hours; 4. Steamed then soaked and 5. Soaked then steamed. Post treatment hays were tested for water soluble carbohydrates, bacteria and mould contents. Differences between means were determined using ANOVA and least significant difference with hay (5), bale (3) and treatment (5) as fixed factors, thus n = 75. Protein and ash proportions were unaltered in any of the treatments. Soaked, steamed then soaked and soaked then steamed treatments were all equally effective at reducing water soluble carbohydrates, with significantly (P<0.05) lower mean contents (79–83 g/kg DM) compared with 126 and 122 g/kg dry matter (DM) for dry and steamed respectively. Steamed and soaked then steamed had significantly (P<0.05) less bacteria (1.04×10^3^ and 4.9×10^2^ CFU/g DM) compared with soaked which increased CFU/g DM from 6.0×10^4^ in dry hay up to 3.5×10^5^. Mould contents CFU/g DM were significantly (P<0.05) reduced by steaming (2) and soaking then steaming (1.9) but no difference was seen between dry (1148), soaked (692) or steamed then soaked (501). Soaking for 9 hours followed by steaming for 50 minutes in the Haygain steamer was the most effective method for reducing water soluble carbohydrates and microbial contamination in hay. Soaking or steaming+soaking lowered water soluble carbohydrates but significantly reduced the hygienic quality of the hay which could potentially compromise the health of the horse.

## Introduction

Grass hay is still the most common fodder fed to stabled horses in the UK [1] and globally [2]. Hay is classified into two categories, seed hay, which is generally composed of one or two specially sown grass species and meadow hay, which is a mixture of different grasses and other herbage in permanent pasture. The nutrient quality of both is primarily influenced by the stage of growth at harvest as well as the mixture of different grasses and other herbage in permanent pasture. Nutrient quality, particularly the content of water soluble carbohydrates (WSC) is strongly influenced by weather conditions at harvest, while the protein content is more influenced by physiological age and soil nutrient status [3]. However, even mature hay conserved from a stressed pasture, e.g. that was subjected to drought, can have high WSC content [4]. As ingestion of high levels of WSC are implicated in insulin resistance [5], equine metabolic syndrome (EMS) [6] polysaccharide storage myopathy [7] (PSSM) and laminitis [8], such hays would be unsuitable for animals at risk of these conditions. Alternatively it has been recommended that hays or forage replacers with a WSC level <100 g/kg DM should be fed to such animals [9]. Many hays, however, exceed this level, with common levels ranging between 100 and 310 g/kg DM [10]. In an attempt to reduce WSC levels, owners soak hay for long periods, often for 12 hours or more. While soaking does decrease WSC levels, Longland and colleagues [11] demonstrated that the efficacy of this procedure varies considerably, with WSC losses ranging from 9 to 54% after a 16-hour soak in water at 16°C. Furthermore, the extent of these losses did not correlate with grass species, or WSC content, so it is not possible to predict how soaking will affect the WSC loss from any individual hay.

Soaking hay for extended periods leaves post-soak liquid that has been reported to have a biological oxygen demand (BOD) of nine times that of raw sewage [12] and as such, should be disposed of carefully and not poured down storm drains. Additionally other studies [13]–[14] have reported that soaking increases the bacterial content of hay. This increase could raise the bacterial concentration above the upper safe limit of 20 µg/g stated by Kamphues [15]. Ingestion of feed-associated bacteria is accompanied by ingestion of significant levels of lipopolysaccharides. These lipoglycan compounds are found in the outer membrane of Gram-negative bacteria and can potentially elicit undesirable strong immune responses in animals [16].

Some horse owners in the UK are now using commercial steamers to improve the hygienic quality of hay. Poor fodder hygiene has been linked to the allergic respiratory condition Recurrent Airway Disorder (RAO) in horses. RAO is characterised by airway bronchoconstriction, neutrophilic inflammation and excessive mucus production which results in reduced dynamic lung compliance, increased pulmonary resistance and reduced performance [17]. Palliative treatment by long-term administration of corticosteroids and bronchodilators contravenes competition rules, is expensive, and the former is considered a potential risk factor in induction/exacerbation of laminitis. So to help maintain the animal in an asymptomatic state, treatment of RAO to-date has been confined to reducing the dust/animal interaction [18]. Steaming for 50 minutes in the Haygain1000 (HG 1000) (Propress Equine Ltd, Hungerford, UK) has been shown to significantly reduce respirable particles and microbiota in hay [19]–[20]. WSC losses post steaming measured across 30 different UK hays [20] averaged 18% but were as variable (2−54%) as those reported by Longland [11] for soaking. The inconsistency of these processes means that neither soaking nor steaming alone can be relied upon as strategies for consistently reducing the WSC content in hay.

The aim of this study was to measure the effect of soaking, steaming and a combination of both treatments on the nutrient content, in particular WSC and microbial contamination of 5 different UK conserved hays intended for horses. The study revealed that soaking alone or after steaming increased the bacterial content of hay while the combination of soaking followed by steaming was the most effective method for reducing both WSC and microbial content in hay intended for horses.

## Methodology

### Forage

Five different types of hay, made in the summer of 2012, were sourced from UK hay merchants in Wiltshire, Berkshire and Devonshire. Three replicate bales of each of the following types were purchased: Timothy mix meadow hay, Italian Rye Grass *(Lolium multiflorum*) seed hay, pure Timothy (*Phleum pratense*) seed hay, medium-cut Meadow hay and late-cut Meadow hay. Post-purchase, all bales were clearly labelled and stored off the ground on pallets in a purpose built, fully enclosed, wooden building at the Royal Agricultural University. Each hay type in succession underwent the following preparation. The three replicate bales were opened and thoroughly mixed on a clean plastic sheet in a glass house. Approximately 2 kg of hay was placed into each of 15 small-holed hay nets. Hay nets were then put into purpose made, pre-labelled, polyester hay bags, 5 hay nets per bag, and stored for approximately one week in a dry, non-heated, well ventilated glass house prior to treatment.

### Treatments

Three replicate hay nets for each type of hay were individually subjected to one of the following treatments. 1. Dry (D) where no additional treatment was applied to the hay; 2. Steamed (S) for 40 minutes in the Haygain 600 hay steamer (Propress Equine Ltd, Hungerford, UK); 3. Soaked (W) by total immersion in 30 litres of clean tap water at 16°C for 9 hours, then hung up to drain for 10 minutes; 4. Steamed for 40 minutes in the Haygain 600 steamer and then soaked (SW) for 9 hours by total immersion in 30 litres of clean tap water at 16°C; 5. Soaked for 9 hours in 30 litres of clean tap water at 16°C, allowed to drain for 10 minutes and then steamed (WS) in the Haygain HG 600 hay steamer for 40 minutes. Each of the five different types of hays were treated as above.

Post- treatment the hay was mixed and two sub-samples were taken. Sub- sample 1 (approximately 10 g) was placed into a sterile plastic bag and placed in a laminar-flow cabinet (Bassaire, Duncan Rd, Swanwick, Southampton), for later microbial analyses. Sub- sample 2 (500 to 800 g, depending on the post treatment DM content), was weighed onto a pre-weighed foil tray and placed in a force-draught oven at 60°C and dried until constant weight was achieved. The sample was then milled using a 1093 Cyclotec Sample Mill (Foss Sweden) and 50 g of the dried, milled sample was retained and stored in sterile plastic tubes (VWR, UK) for subsequent WSC, CP and ash analyses.

An eighty millilitre sample of liquid was taken from the post-soak tubs and the post-steam trays that collected the condensation from the steamer for assessment of TVC and mould concentrations in the post - treatment liquids.

### Microbial analyses

The 10 g post- treatment sub-sample was roughly chopped with scissors, (previously wiped with ethanol, and allowed to dry) and thoroughly mixed. A one gram sub-sample was then weighed into a sterile plastic bag (Seward BA6040) to which 79 ml of peptone saline solution (MRD) was added. The bag was then placed into a Lab Blender 80 model (Steward Laboratory, Blackfriars Rd, London). The mixture was then ‘blended’ for 2 minutes in order to wash mould and bacteria from the hay into the solution as per instruction manual for 3M petrifilms (3M Microbiology, 2013). One millilitre of the blended solution was placed into a sterile screw-cap tube (VWR, UK) pre-loaded with 9 ml MRD. Serial dilutions were prepared to 10^−6^. A 1 ml sample was then taken from 10^−2^, 10^−4^ 10^−6^ dilutions and separately placed onto pre-labelled 3M Aerobic TVC 20 cm^2^ petrifilm, and 3M Yeast and Mould 30 cm^2^ petrifilm (3M Microbiology, Carl-Schurz-StraBe 1, Germany). Petrifilms are a sample ready, culture medium containing nutrients, a cold water soluble gelling agent, a tetrazolium indicator for the TVC films, and antibiotics for determination of yeasts and moulds. Six petrifilms (three TVC and three yeast and mould) were prepared for each sample. TVC samples were incubated for 3 days at 32°C, yeast and mould films were incubated for 5 days at 20°C.

Samples of the liquid from the post-soaking tubs and steamer condensate were processed according to the protocol described above, except that 1 ml of this fluid was directly placed into 79 ml of MRD, thoroughly mixed before serial dilutions to 10^−6^ were prepared.

### Microbial enumeration

Colony numbers were enumerated using an illuminated magnifier. For TVC films, all vital stained colonies were counted. When colony numbers were particularly dense and small and >100 per film, three representative 1 cm squares were counted. The average was determined, and scaled up 20-fold as an estimation of the count per film. Mould colonies were identified as described by Moore-Colyer and Fillery [13] by their large flat areas with diffuse edges and dark centres, while yeast colonies were small, tan colour and had no clear centre. Fungal and mould colonies were counted as total colonies grown per film, or when numbers were greater than 200 representative squares were counted and scaled up 30-fold as an estimation of the count per film.

### Nutrient analyses

Crude protein was measured using the Kjeldahl process. Acid Detergent Fibre plus ash were measured as described by the Association of Official Analytical Chemists [21]. WSC were measured by the Phenol-sulphuric acid method [22].

### Data analyses

Differences in nutrient contents, TVC and mould concentrations from this Randomised Block Experiment were determined using analysis of variance (ANOVA), with hay (5), bale (3) and treatment(5) as fixed factors; thus n = 75. Differences between means were calculated using least significant difference (LSD) test where LSD = t _(error df)_ × s.e.d, on log 10 transformed data (Genstat 15, 2013) [23] as per the procedure for right-handed skewed data [24]. Results for nutrient composition were expressed as g/kg on an as fed and DM basis, while those for TVC and mould were expressed as geometric mean colony forming units (CFU)/g on an as fed and DM basis, as this value approximates closely to the median [24] which is widely accepted to be the most accurate expression of the distribution of the CFU in the samples Differences between microbial contamination of the post-soak and post- steam liquid were also determined using analyses of variance, with hay (5), bale (3) and treatment (4) thus n = 60; and LSD test as detailed above.

## Results

### Nutrient composition and microbial content in hay

The five types of hay used in this study were a range of seed and meadow hays that are commonly fed to horses in the UK. As detailed in Table 1, the nutrient and microbiological content of the hays showed that they varied in their water soluble carbohydrate (WSC) content from 201 g/kg DM in the Timothy mix hay down to 44 g/kg DM in the more stemmy late-cut Meadow hay. The opposite occurred for crude protein (CP) contents which were lowest in the Timothy mix hay at 39 g/kg DM and highest in the late-cut Meadow hay at 80 g/kg DM. Bacterial contents, all expressed on an as fed basis, varied across all the hays from 120 CFU/g in the Italian rye grass (IRG) hay to 3.0×10^6^ CFU/g in the late-cut meadow hay, while mould levels ranged from 0 CFU/g in the medium-cut meadow hay to more than 4.0×10^6^ CFU/g in the IRG. These levels did not appear to be associated with any particular nutrient content or maturity level. Timothy hay showed high abundances of bacteria and mould, based on the classification used by Adams ^(25)^, whereas the other four hays had either high TVC or high mould contents.

**Table 1:** Mean nutrient contents (g/kg DM), bacteria and mould colony forming units (CFU/g) of 5 different types of UK conserved hay.

	Timothy mix	Italian rye grass	Timothy	Meadow hay (medium)	Meadow hay (late)
Dry matter	900	890	890	870	810
Water soluble carbohydrates	201	91	140	153	44
Crude protein	39	64	60	73	79
Acid detergent fibre	210	320	250	220	280
Ash	10	10	3	10	33
Bacteria (cfu/g)	233833	120	503833	448071	3000000
Mould (CFU/g)	80	4685666	503834	0	1732

### Effect of treatment on nutrient composition

The soaking and steaming treatments significantly (P<0.05) increased the moisture content of all the hays. The moisture content of the original hays averaged 13% whereas after steaming this was increased to 27% and after any of the soaking treatments from 70 to 73%. Thus the DM content for each of the post-treated hays was D = 87%, S = 73%, W and SW = 27% and WS = 30%.

Table 2 shows the different treatment effects on the nutrient content of the tested hays expressed on a g/kg DM basis. Soaked (W) as well as the SW and WS treatments reduced (P<0.05) WSC levels from 12.6±5.5% to 8.1±4.4%, while S alone had little effect. Numerically the greatest mean loss across all of the hays in WSC content occurred with the WS treatment (37% reduction) but this was not significantly different from the loss seen with W or SW (both 34%), suggesting that all 3 methods that included a soaking step were equally effective at reducing WSC. The range of WSC leaching from individual hays across the 5 treatments is shown in Table 3. For W, SW and WS treatments WSC loss was greatest from Timothy> IRG> Meadow medium> Meadow late> Timothy mix. Steaming (S) produced a different order of WSC loss with Meadow late> Timothy> Timothy mix> IRG> Meadow medium. The loss of WSC across all the soaking treatments resulted in enrichment of insoluble acid detergent fibre (ADF), which significantly (P<0.001) increased as a proportion of total hay DM. While proportionally the CP content remained unaltered on a g/kg DM basis, the overall loss in DM indicates that some loss of CP must have occurred.

**Table 2:** Mean crude protein (CP), acid detergent fibre (NDF), water soluble carbohydrates (WSC) and ash contents (g/kg DM) of five different UK conserved hays when subjected to five different wetting treatments.

	Dry (D)	Steamed (S)	Soaked (W)	Steamed+soak (SW)	Soak+steam (WS)	s.e.d	Sig
CP	63	63	62	61	62	1.17	NS
ADF	259^a^	265^a^	298^bc^	285^b^	310^c^	9.98	0.001
WSC	126^b^	122^b^	83^a^	83^a^	79^a^	4.66	0.001
Ash	13	17	15	15	14	1.66	NS

abcd Values in the same row not sharing common superscripts differ significantly (p<0.001).

**Table 3:** Mean contents of water soluble carbohydrate (WSC)(g/kg DM) in 5 different types of UK hay when subjected to 5 different wetting treatments.

	Timothy mix	Italian rye grass	Timothy	Meadow hay (medium)	Meadow hay (late)	Mean (± SD)
Dry (D)	201	91	140	153	44	126±5.47
Steamed (S)	194	88	135	151	41	122±5.34
Soaked (W)	155	55	66	104	32	83±4.32
Steamed+soaked (SW)	166	50	76	92	31	83±4.67
Soaked+steamed (WS)	152	51	69	94	29	78.9±4.27

### Microbial contamination

As shown in Table 4 all treatments resulted in a minor numerical decrease in mould CFU/g on an as fed and DM basis. W and SW treatments however, did not produce significantly lower microbial CFU/g compared with that for the dry hay. Steaming (S) and WS, however, resulted in a significant reduction (P<0.05) in CFU/g (2 CFU/g) on both an as fed and DM basis.

**Table 4:** Geometric mean numbers (CFU/g) of bacteria (TVC) and mould in five UK conserved hays when subjected to five different wetting treatments.

	Dry (D)	Steamed (S)	Soaked (W)	Steamed+soaked (SW)	Soaked +steamed(WS)	s.e.d	Sig
TVC/g as fed	51286^bc^	741^a^	186209^c^	10233^b^	309^a^	3.21	0.001
TVC/g DM	60256^bc^	1046^a^	354813^c^	17783^b^	490^a^	3.21	0.001
Mould/g as fed	1047^b^	2^a^	380^b^	288^b^	1.8^a^	3.53	0.001
Mould/gDM	1148^b^	2^a^	692^b^	501^b^	1.9^a^	3.53	0.001

abc Values in the same row not sharing common superscripts differ significantly (P<0.001).

Steaming (S) as well as WS significantly reduced TVC contents by 93 to 99% respectively, compared with all other treatments. Soaking alone (W) significantly (P<0.05) increased the levels to four-times the amount of bacteria found in the dry hays.

The microbial counts of the post-treatment liquids confirm the trends of bacteria and mould contamination found in the post-treatment hay samples (Table 5). Steaming (S) reduced the bacteria by 99.7% and mould by 96.4% and WS reduced bacteria by 99.1% and mould by 93.1% compared with that found in the post W liquid. The SW treatment did not significantly reduce microbial contamination compared with the W treatment, demonstrating the stimulating effect of water on the growth of microbes.

**Table 5:** Geometric mean numbers (CFU/ml) of bacteria (TVC) and mould in the effluent after soaking, steaming and a combination of both applied to five different UK hays.

	Steamed (S)	Soaked (W)	Steamed+soaked (SW)	Soaked +steamed (WS)	s.e.d	Sig
TVC/ml	110^a^	29512^b^	8128^b^	288^a^	3.04	0.001
Mould/ml	1.23^a^	26.3^b^	18.62^b^	2.09^a^	2.13	0.001

abc Values in the same row not sharing common superscripts differ significantly (P<0.001).

## Discussion

### Nutrient content

The five hays tested in this study had typical WSC and CP contents for UK hays [20]–[26] and reflect the quality of hay fed to horses across the UK. The late-cut meadow hay had a low DM content of 81%, which is below the widely accepted recommended level of 85% DM for storing hay [27]. As both bacteria and mould require moisture to proliferate, the low hay DM plus the warm damp conditions that prevailed in the 2012 UK hay-making season, would have contributed to higher microbial counts in that particular batch of hay. Additionally, bacteria and moulds use nutrients in the hay for growth; hence hays with high microbiota can also have low WSC and CP levels as demonstrated in this study.

Visual assessment revealed that the Italian ryegrass, closely followed by the late-cut meadow hay, contained high ratios of stem: leaf and so were the most fibrous of all the 5 hays tested. This result was confirmed by their high ADF, low WSC and low CP contents. In terms of nutrient supply the mid-cut meadow hay contained the most WSC and CP. However, none of the hays used in this study would have supplied the recommended CP requirements for a horse on a maintenance diet [28] if fed as the sole diet at approximately 2.5% BW/day.

### Microbial contamination

Although there were no visible signs of mould or bacteria in any of the hays studied, mould and bacterial spores were present in all the hays. Hays that had the highest bacterial content in this study had the lowest mould contamination and vice versa. Seguin [29] reported that the variety of fungal species and contamination level could be attributed to an ecological niche which in turn was influenced by harvest date. Although no published information could be found on the relationship between mould and bacteria contamination of hay it is likely that an ecological niche was again the main factor that gave competitive advantage of one type of micro-organism over the other. Mould levels were particularly high (5.0×10^5^ to >4×10^6^) in both the single species hays (IRG and Timothy) which agree with previously published data^(13)^ of levels in the region of 1.1×10^6^ CFU/g in pure PRG hay. The lower levels detected in both meadow hays and the Timothy mix hay, demonstrate the variability in hygienic quality of UK hays and also reflect the poor hay-making season of 2012. Those farmers who were fortunate enough to catch the few dry days would have made ‘cleaner hay’ than crops that were rained upon or baled too early in an attempt to ‘beat’ poor weather. This demonstrates the importance of ensuring that hay purchased for horses has been conserved under good, dry conditions.

### Wetting treatments

Steaming increased the moisture content across all of the hays 2 fold and soaking by over 5-fold. Two previous studies have shown that steaming hay increased palatability compared with soaked hay [30] and haylage [31]. The increased palatability may be partly due to the increase in water content and softness of the hay which appeals to the horse’s highly developed organoleptic senses [32]. A study by James and Moore-Colyer [20] reported that in 30 different hay samples collected across the UK, steaming reduced the WSC content by an average of 18% while protein and mineral contents remained unaltered. This reduction in WSC content would make the hay less ‘sweet’ to taste, but the small increase in water content and softness of the hay, coupled with the appealing aroma may have enticed the horses to choose steamed hay over the more acidic haylage and the very wet post-soaked hay used in their study.

None of the wetting treatments applied to the five hays in this study caused a reduction in the proportion of DM represented by total minerals (as measured by ash content) nor of CP. However, as the CP proportion remained the same post-treatment as in the dry hay, some loss of soluble protein must have occurred. Indeed individual hays, namely the IRG and Timothy showed loss of CP while the more mature Meadow Late cut increased in proportion post-treatment. These results reflect the proportion of soluble and cell-wall bound protein within the different types of hay. As with previously reported work, these losses seemed to be very variable with some studies reporting no loss [19-33-34] while others report significant losses [12]. A more recent study by Martinson [35] involving soaking Orchardgrass and Alfalfa hays at different temperatures and durations, found that the losses of both CP and minerals were highly variable. The variability of nutrient losses reported in all of these studies could be due to variety of grass species, stage of growth at harvest and edaphic conditions. In mature crops if the protein is cell-wall bound then leaching of this nutrient is likely to be low, while leafy forage with a higher soluble protein content may indeed suffer larger losses of CP after soaking.

The mean loss of WSC with steaming (S) was only 3% with a range of 1.4 to 6.9% for Meadow medium cut and Meadow late cut hays respectively. These losses are notably lower than the 18% noted across the 30 UK hays [20] when using the Haygain steamer and the 12% recorded by Earing [36] when steaming moderately mouldy alfalfa-orchard grass hay in a Happy Horse hay steamer. In the same study no WSC loss was reported from the same species hay, cut at the same stage of growth and with a similar nutrient profile but which they classified as having low mould content. All three studies demonstrate that loss of WSC from steaming hay is influenced by individual hay characteristics and cannot be predicted from species, stage of growth or hygienic quality.

Soaking (W) caused an average loss of water soluble carbohydrates (WSC) across all the hays tested in this study of 34% (range of 23% Timothy mix, to 53% Timothy pure) and a concomitant, proportional rise in Acid Detergent Fibre (ADF) of 15%. Although WS produced the highest WSC losses of 37% (range of 24% Timothy mix to 51% Timothy pure), this was not significantly different from the W and SW treatments. However, in agreement with the study of Longland [3] the range of WSC losses noted here was large with the lowest loss across W, SW and WS treatments occurring from the Timothy mix hay at 17% and the greatest losses from Timothy pure hay at 53%. These results confirm previous findings with regard to WSC [3] and CP [33] losses showing that soaking has a very variable effect on nutrient loss from different hays.

### Microbial counts post treatment

The average bacterial contamination across all five hays was increased 5 fold when the hays were soaked for 9 hours in water. These results are in agreement with earlier findings of Moore-Colyer and Fillery [13] who also reported a 1.5- fold increase in bacteria counts when soaking hay for 10 minutes. These results show that submerging hay in water can cause rapid and extensive proliferation of bacteria. The bacterial concentrations in the post-soak effluent indicated that some bacteria were washed off the hay during soaking and were able to survive and proliferate in the soak water, which certainly contained leached carbon and possibly some soluble protein [12]. This level of contamination could increase the amount of bacteria ingested to above the recommended safe limit of 20 µg/g [15]. Ingestion of feed-associated bacteria will also mean the ingestion of significant levels of lipopolysaccharides, (the lipoglycans found in the outer membrane of Gram-negative bacteria), which can elicit undesirable strong immune responses in animals. Furthermore, recent work (Xu, pers.com) has shown that potentially pathogenic bacteria such as *Acinetobacter iwoffi,* are present in dry hay and the proliferation of any of these by the addition of water would be highly undesirable.

Steaming (S) and soaking+steaming (WS) on the other hand reduced (P<0.001) the CFU/g of bacteria by 98–99%. This indicates that soaking in an attempt to cause WSC loss in hay is an acceptable procedure if it is followed by high-temperature steaming to kill microorganisms that may have proliferated during the soaking process. However, as Taylor and Moore-Colyer [14] showed, the steam must fully penetrate all the hay and maintain a temperature of >90°C for a minimum of 10 minutes for all the mould and most of the bacteria to be killed. Insufficient steaming, i.e., releasing steam into a non-insulated container does not reach the necessary high temperatures throughout the hay to kill most of the bacteria. Partial steaming therefore has a similar effect to soaking causing bacteria proliferation rather than achieving a reduction and thus could increase potentially pathogenic bacteria, especially *Thermoactinomyces spp* which thrive at temperatures of between 18–40°C.

The steamed+soaked (SW) treatment did not reduce the bacterial contamination of the hays. Steaming alone has been reported to kill between 60% [14] and 99% [13] of bacteria present in hay. The bacteria remaining in the post-steamed hay are likely to be thermo- stable spore-formers that rapidly proliferate once they become hydrated during soaking. Potentially allergenic *Actinobacteria* such as *Saccharopolyspora rectivirgula* and *Thermoactinomyces vulgaris* have been documented to be present in hay [37]–[38] and these organisms can withstand temperatures over 100°C for 4 hours so are unlikely to be killed during steaming. Proliferation of any of these bacteria by soaking after steaming is therefore highly undesirable.

Effluent values from the post-steamed samples showed a 99% reduction of bacteria compared with that seen in the post-soak fluid. This is in agreement with previous studies [13], [20] which also demonstrated that steaming kills a high proportion of bacteria present in hay. No identification of bacterial species was undertaken in this study, but as this and earlier studies indicate, some bacteria do survive the high-temperature steaming so further work is required to identify if these bacteria are potentially pathogenic.

Steaming (S) or Soaking+steaming (WS) reduced mould contamination by>99% across the range of low, medium and highly contaminated hays tested in this experiment. These results are in agreement with previously published results when steaming hay^(20, 13)^. It would seem therefore; that the mechanism of injecting high-temperature steam *via* a spiked manifold into the centre a bale is a highly effective way of reducing mould contamination in hay. Soaking hay and the treatment where steaming was followed by soaking did not significantly reduce the mould contamination across all the hays, The results on soaking are in agreement with those of Moore-Colyer and Fillery [13] and Taylor and Moore-Colyer [14] who also found that soaking was significantly less effective than steaming at reducing mould in hay.

Post-soak liquid values indicated that a small amount of viable mould spores were present in the post-steamed fluid. However, at only to1 to 2 CFU/ml it is possible that this contamination occurred from airborne spores settling onto the surface of the fluid collected at the base of the steamer.

### Feeding treated hay

Although further work is needed to confirm the potential clinical relevance of this, the loss of dry matter, largely in the form of WSC from the treated hays meant that there was a concomitant rise in the proportion of insoluble acid detergent fibre (ADF). This is in agreement with the study of Pagan [39] who also noted an increase in ADF content after soaking and steaming Timothy hay. However, they also reported that the proportional increase in fibre did not negatively affect *in vivo* digestibility values for DM, CP, fat, ADF, NDF, WSC, NSC nor ash. Mineral levels as measured by the proportion of ash present were not significantly affected by any of the wetting treatments, although as there was a loss in DM some mineral loss may have occurred. However, ash is a very imprecise indication of mineral content, thus owners should have hay analysed post-treatment for the content of individual minerals in order to accurately balance the micro-nutrient profiles of the diet. Overall proportions of protein remained constant but given there was a loss in DM, on an individual hay basis some lost more soluble CP than others. Several of the hays used were mature with a high proportion of stem: leaf, so it is likely that most of the CP was cell-wall bound and therefore insoluble. The hays used in this study were typical UK hays and it is worth noting that while soaking may leach variable amounts of CP (depending in part on the proportion of soluble: insoluble CP), the fact that even when un-soaked the majority is cell-wall bound will render it largely unavailable to the horse. Mature hay therefore, cannot be relied upon to meet the CP requirements of a horse, fed at 2.5% BW of DM per day [28].

Results from this study indicate that WS caused the greatest loss of WSC from hay and the largest reduction in bacterial and mould content. Thus, this treatment produced clean and comparatively low sugar hay suitable for feeding to obese and laminitis - prone horses and ponies. Steaming alone produced hygienically clean hay but did not reduce the WSC content to below the suggested threshold of 100 g/kg for feeding to horses with metabolic disorders. The commonly used practice of soaking hay does result in losses of WSC although these are variable and prolonged soaking also results in a substantial increase in bacterial contamination. Therefore, soaking hay for prolonged periods without further treatment compromises the hygienic quality of the hay, which could have clinical consequences but more work is needed to confirm this.

## Conclusion

Soaking hay for 9 hours followed by a 50 minute high-temperature steam in a Haygain steamer was the most effective and consistent method for reducing WSC and microbial contamination of hay for horses. While soaking hay or steaming followed by soaking can be an effective way of reducing WSC content, the losses were highly variable (range of 0–53% loss) and significantly increased the bacterial content, compared to the original, dry, untreated hay, thus reducing the hygienic quality of the hay.
